# Exploring the genetics of nephronophthisis and Joubert Syndrome…more than monogenic cystic renal diseases?!

**DOI:** 10.1186/2046-2530-1-S1-P103

**Published:** 2012-11-16

**Authors:** RJ Simms, M Hynes, JA Sayer

**Affiliations:** 1 Institute of Genetic Medicine, Newcastle University, Newcastle-upon-Tyne, UK; 2 Department of Renal Medicine, Freeman Hospital, Newcastle-upon-Tyne, UK

## Background

Understanding the pathogenesis of the autosomal recessive cystic kidney disease, nephronophthisis (NPHP) remains a challenge. NPHP is associated with extra-renal disease in 10-15%, including abnormal eye and cerebellar development, this combination of problems is called Joubert Syndrome, JS. NPHP and JS are ciliopathies because the encoded proteins of all mutated genes are found in primary cilia/associated architecture. NPHP and JS are genetically heterogenous, with mutations in a single gene such as *NPHP6*, *AHI1* or *CC2D2A* being sufficient to cause disease. Although homozygous mutations are identified in most cases, some patients have an additional heterozygous mutation in another gene, leading to hypotheses of epistasis modifying the clinical phenotype.

## Objectives

We aim to use zebrafish, in whom *nphp6*, *ahi1* and *cc2d2a* are highly conserved, as a model organism, to evaluate potential genetic interactions and the influence this may have on the development of NPHP and JS.

## Methods

Splice blocking antisense morpholino oligonucleotides (MOs) directed towards *nphp6*, *ahi1* and *cc2d2a* were injected individually, and in combination, into 1-4 cell stage wild type zebrafish embryos. Embryos were phenotyped using light microscopy at 72 hours post fertilisation (hpf).

## Results

Each MO individually induces a morphant phenotype including curly tail, cardiac oedema, hydrocephalus, pronephric cysts, abnormal eye and ear development. Co-injection of low dose *ahi1* and *nphp6* MOs or *ahi1* and *cc2d2a* MOs leads to synergy of the morphant phenotypes.

## Conclusion

The synergistic increase in the morphant phenotype following combined knockdown of *ahi1* and *nphp6* or *ahi1* and *cc2d2a* in zebrafish implicates a genetic interaction.

